# *Lignipirellula cremea* gen. nov., sp. nov., a planctomycete isolated from wood particles in a brackish river estuary

**DOI:** 10.1007/s10482-020-01407-4

**Published:** 2020-04-01

**Authors:** Stijn H. Peeters, Sandra Wiegand, Nicolai Kallscheuer, Mareike Jogler, Anja Heuer, Mike S. M. Jetten, Christian Boedeker, Manfred Rohde, Christian Jogler

**Affiliations:** 1 Department of Microbiology, Radboud Universiteit, Nijmegen, The Netherlands; 2 Institute for Biological Interfaces 5, Karlsruhe Institute of Technology, Eggenstein-Leopoldshafen, Germany; 3 Department of Microbial Interactions, Institute of Microbiology, Friedrich Schiller University, Jena, Germany; 4 Leibniz Institute DSMZ, Brunswick, Germany; 5 Central Facility for Microscopy, Helmholtz Centre for Infection Research, HZI, Brunswick, Germany

**Keywords:** Aquatic bacteria, *Planctomycetes*, *Pirellulaceae*, Baltic Sea, Budding, Wood particles

## Abstract

A novel planctomycetal strain, designated Pla85_3_4^T^, was isolated from the surface of wood incubated at the discharge of a wastewater treatment plant in the Warnow river near Rostock, Germany. Cells of the novel strain have a cell envelope architecture resembling that of Gram-negative bacteria, are round to pear-shaped (length: 2.2 ± 0.4 µm, width: 1.2 ± 0.3 µm), form aggregates and divide by polar budding. Colonies have a cream colour. Strain Pla85_3_4^T^ grows at ranges of 10–30 °C (optimum 26 °C) and at pH 6.5–10.0 (optimum 7.5), and has a doubling time of 26 h. Phylogenetically, strain Pla85_3_4^T^ (DSM 103796^T^ = LMG 29741^T^) is concluded to represent a novel species of a novel genus within the family *Pirellulaceae*, for which we propose the name *Lignipirellula cremea* gen. nov., sp. nov.

## Introduction

*Planctomycetes* is a phylum of bacteria which were once thought to have several exceptional eukaryote-like traits (Devos et al. 2013; Devos and Reynaud 2010; Fuerst and Sagulenko 2011; Fuerst and Webb 1991; König et al. 1984; Lindsay et al. 1997; Lonhienne et al. 2010). These have since been re-examined and re-interpreted (Acehan et al. 2013; Boedeker et al. 2017; Jeske et al. 2015; Jogler 2014; Jogler et al. 2011; Jogler and Jogler 2013; Neumann et al. 2014; Rast et al. 2017; Rivas-Marin et al. 2016b; Rivas-Marín and Devos 2018; Santarella-Mellwig et al. 2013; van Teeseling et al. 2015). The cell envelope architecture of Planctomycetes was shown to resemble that of Gram-negative bacteria (Boedeker et al. 2017; Devos 2014). The phylum *Planctomycetes* is part of the PVC superphylum, along with *Verrucomicrobia*, *Chlamydiae* and others (Wagner and Horn 2006). Members of the phylum can play major roles in biogeochemical cycles (Peeters and van Niftrik 2018; Strous et al. 1999; Wiegand et al. 2018), are ubiquitous and are found in high abundance on algal surfaces (Bengtsson and Øvreås 2010; Bengtsson et al. 2012; Bondoso et al. 2014, 2015, 2017; Lage and Bondoso 2014; Vollmers et al. 2017), on which they probably metabolise complex sugars (Frank et al. 2014; Jeske et al. 2013; Lachnit et al. 2013; Wiegand et al. 2018).

Major scientific interest is dedicated to the interesting cell biology of *Planctomycetes*. For example, they lack many otherwise essential cell division genes, including the canonical *ftsZ* (Jogler et al. 2012; Pilhofer et al. 2008; Wiegand et al. 2020). Even so, some Planctomycetes are capable of performing cell division by binary fission, while others divide by budding (Rivas-Marin et al. 2016a; Wiegand et al. 2020). Members of the orders *Planctomycetales* and *Pirellulales* also perform a lifestyle switch between sessile mother cells and motile daughter cells (Jogler et al. 2011). Most Planctomycetes feature unusual crateriform structures on their cell surfaces. Their periplasm can be enlarged to form invaginations into the cytoplasm. They are potential producers of small molecules with interesting bioactivities (Graça et al. 2016; Jeske et al. 2016; Wiegand et al. 2018), possess many giant genes (Kohn et al. 2016), and belong to the bacterial phylum with the highest number of predicted genes with unknown function (40–55% of the annotated proteins) (Bordin et al. 2018; Overmann et al. 2017; Wiegand et al. 2018).

In this study, we isolated strain Pla85_3_4^T^ from wood particles floating in the estuary of the Warnow river next to a wastewater treatment plant discharge and close to the Baltic Sea. In phylogenetic analyses, the strain clusters within the family *Pirellulaceae*, which comprises most of the described members of the order *Pirellulales*. In the past, Planctomycetes have been found on decomposing wood in natural temperate forests by 16S rRNA gene analysis (Tlaskal et al. 2017), but to date only one other Planctomycete, *Singulisphaera mucilagenosa*, has been isolated from (degrading) wood (Zaicnikova et al. 2011).

## Materials and methods

### Isolation and cultivation of the strain

Strain Pla85_3_4^T^ was isolated from submerged wood pellets suspended near the discharge of a wastewater treatment plant in the estuary of Warnow river next to the city Rostock located in Germany (exact location 54.106  N 12.096 E). This location is close to the Baltic Sea and has brackish water. Isolation was performed as described previously (Oberbeckmann et al. 2018; Wiegand et al. 2020). For further investigation, the strain was grown in M1H medium supplemented with *N*-acetyl glucosamine (NAG) and artificial seawater (ASW) (M1H NAG ASW medium) as described previously (Wiegand et al. 2020) and was cultivated at 28 °C under constant agitation at 110 rpm.

### Light microscopy and electron microscopy

Phase contrast microscopy was performed with a Nikon Eclipse Ti inverted microscope with a Nikon DS-Ri2 camera. Cells were immobilised in MatTek glass bottom dishes (35 mm, No. 1.5) using a 1% (w/v) agarose cushion (Boedeker et al. 2017). ImageJ (Rueden et al. 2017) was used to examine cell size by sequentially applying an Otsu threshold, then the watershed function, and finally the count particles function, excluding particles smaller than 0.05 µm.

Field emission scanning electron microscopy was performed as described previously (Boersma et al. 2019). The bacteria were fixed in formaldehyde, washed and placed on poly-l-lysine-coated cover slips. Samples were then fixed in 1% (v/v) glutaraldehyde and washed twice before dehydrating in graded series of acetone [10, 30, 50, 70, 90, 100% (v/v)] on ice. Samples from the last acetone treatment step were brought to room temperature before placing them in fresh 100% acetone. Samples were then subjected to critical-point drying with liquid CO_2_ (CPD 300, Leica). Dried samples were covered with a gold/palladium (80/20) film by sputter coating (SCD 500, Bal-Tec), before examination in a field emission scanning electron microscope (Zeiss Merlin) using an Everhart–Thornley HESE2 detector and an inlens SE detector in a 25:75 ratio with an acceleration voltage of 5 kV.

### Physiological and biochemical analyses

The pH optimum was determined at 28 °C, with buffering agents 100 mM 2-(*N*-morpholino)ethanesulfonic acid (MES) at pH 5 and 6, 100 mM (4-(2-hydroxyethyl)-1-piperazineethanesulfonic acid) HEPES at pH 7, 7.5 and 8, and 100 mM *N*-cyclohexyl-2-aminoethanesulfonic acid (CHES) at pH 9 and 10. Temperature optimum determination was performed at pH 7.5 and temperatures ranging from 10 to 40 °C in steps of 5 °C. Cell densities were inferred from optical densities (λ = 600 nm).

### Genome information and analysis of genome-encoded features

The genome and 16S rRNA gene sequences of strain Pla85_3_4^T^ are available from GenBank (accession numbers MK559988 and CP036433, respectively). Numbers of carbohydrate-active enzymes were obtained from the CAZY database (Lombard et al. 2014). Gene clusters potentially involved in the production of secondary metabolites were determined using antiSMASH 4.0 (Blin et al. 2017).

### Phylogenetic analysis

16S rRNA gene-based phylogeny was computed for strain Pla85_3_4^T^, the type strains of all described planctomycetal species (assessed in January 2020), all isolates recently published (Boersma et al. 2019; Kallscheuer et al. 2019a, b, c, d, 2020; Kohn et al. 2019; Peeters et al. 2019; Rensink et al. 2020) and with an outgroup of strains from outside the phylum *Planctomycetes* but part of the PVC superphylum. The alignment of 16S rRNA genes was made with SINA (Pruesse et al. 2012). Phylogenetic analysis was performed employing a maximum likelihood approach with 1000 bootstraps, the nucleotide substitution model GTR, gamma distribution, and estimation of proportion of invariable sites using GTRGAMMAI (Stamatakis 2014).

The genomes for the genome-based analyses were gathered from GenBank including the sequences for strain Pla85_3_4^T^ recently published (Wiegand et al. 2020). Completeness and contamination of the genome was determined using CheckM v1.0.131 (Parks et al. 2015). The average nucleotide identity (ANI) was calculated using OrthoANI (Lee et al. 2016), the average amino acid identity (AAI) was computed with the aai.rb script from the enveomics collection (Rodriguez-R and Konstantinidis 2016) and the percentage of conserved proteins (POCP) was determined as previously described (Qin et al. 2014). The *rpoB* nucleotide sequences (encoding the β-subunit of RNA polymerase) were taken from the genome annotations and the sequence identities were determined as described (Bondoso et al. 2013). Upon extracting only those parts of the sequences that would have been sequenced with the primer set described by Bondoso et al. (2013), the alignment and matrix calculation was performed with Clustal Omega (Sievers et al. 2011).

For the multi-locus sequence analysis (MLSA), the unique single-copy core genome of all analysed genomes was determined with proteinortho5 (Lechner et al. 2011) with the ‘selfblast’ option enabled. The protein sequences of the resulting orthologous groups were aligned using MUSCLE v.3.8.31 (Edgar 2004). After clipping, partially aligned *C*- and *N*-terminal regions and poorly aligned internal regions were filtered using Gblocks (Castresana 2000). The final alignment was concatenated and clustered using the maximum likelihood method implemented by RaxML (Stamatakis 2014) with the ‘rapid bootstrap’ method and 500 bootstrap replicates. The outgroup consisted of concatenated gene sets of strains from the order *Planctomycetales.*

## Results and discussion

### Phylogenetic inference

**Fig. 1:**
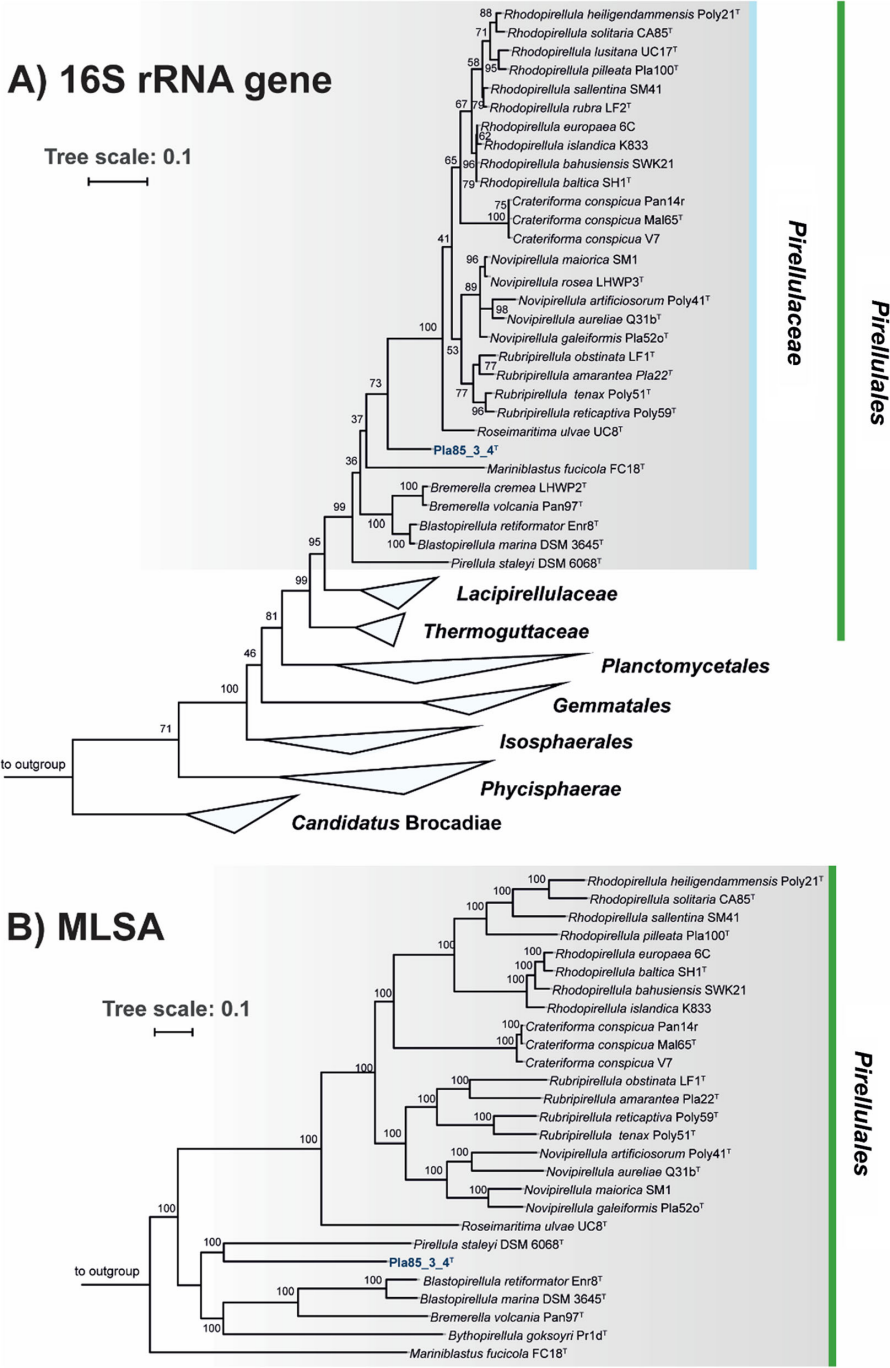
**a** 16S rRNA gene-based phylogenetic tree of described planctomycetal species and the novel isolate Pla85_3_4^T^ indicated in blue. Bootstrap values indicated as a proportion of 1000 re-samplings (in %). The outgroup consisted of three 16S rRNA genes from the PVC superphylum outside the phylum *Planctomycetes*. **b** Whole genome-based MLSA phylogeny, with bootstrap values based on 500 re-samplings, indicated at the nodes (in %). The outgroup consisted of several representatives of the order *Planctomycetales*

**Fig. 2:**
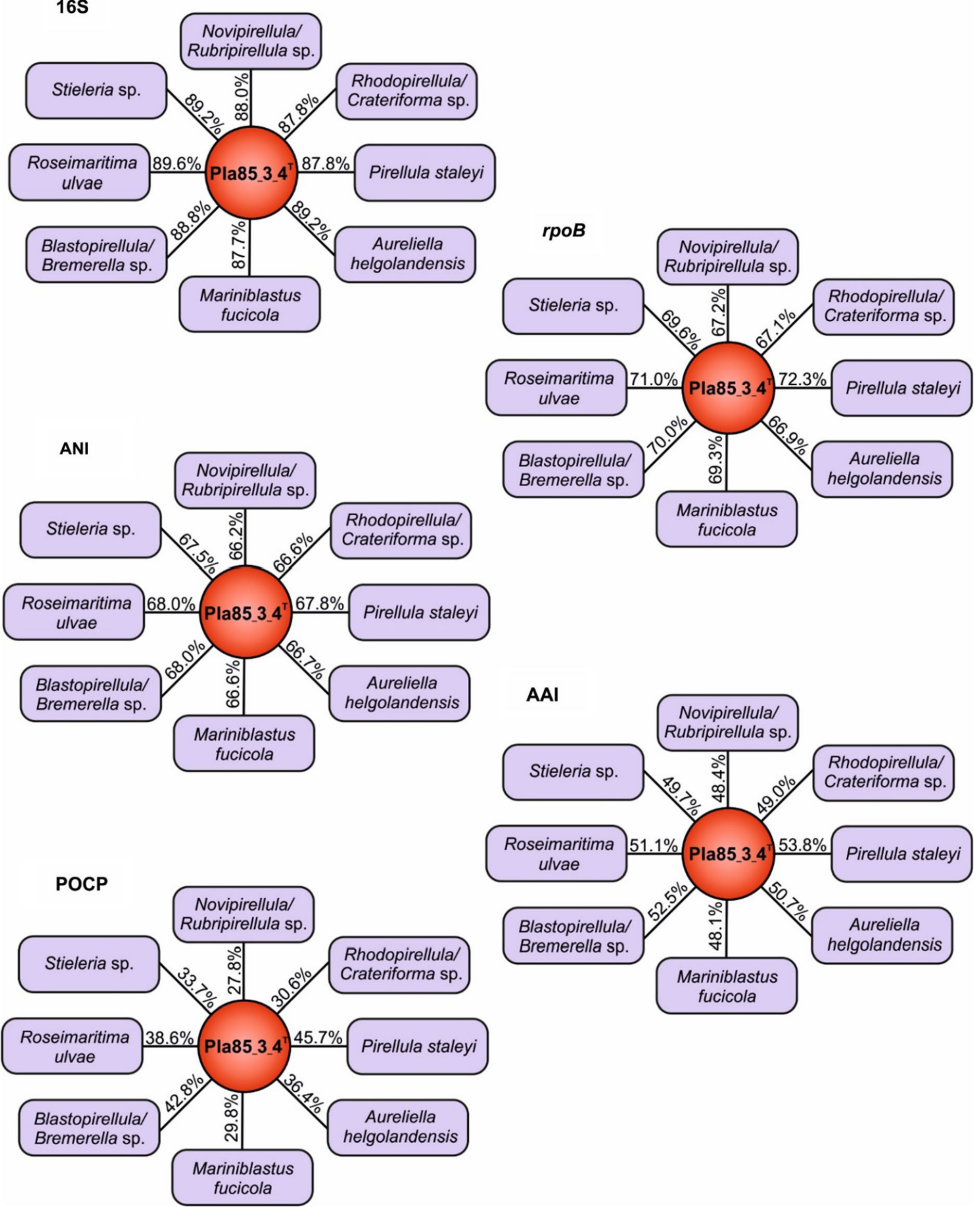
Delineation of strain Pla85_3_4^T^ from known genera in the family *Pirellulaceae*. Methods used: 16S rRNA gene identity (16S), average amino acid identity (AAI), average nucleotide identity (ANI), *rpoB* gene (partial) identity and percentage of conserved proteins (POCP)

Based on 16S rRNA sequence analysis and MLSA, the isolated strain Pla85_3_4^T^ clusters within the family *Pirellulaceae* (Fig. 1). 16S rRNA gene sequence identities to other current genera of this family range from 87.7 to 89.2% (Fig. 2). These values fall below the 94.5% cut-off value for delineating genera (Yarza et al. 2014), indicating that this species is part of a novel genus. To substantiate this claim, other phylogenetic markers such as RNA polymerase β-subunit (*rpoB*) gene similarity (Bondoso et al. 2013), AAI (Konstantinidis and Tiedje 2005) and POCP (Qin et al. 2014) were employed. Comparison of POCP values of strain Pla85_3_4^T^ and the other genera in the family *Pirellulaceae* yielded maximum values between 27.8% and 45.7% (Fig. 2) which are all below the 50% cut-off value for delineation of genera (Qin et al. 2014). Comparison of AAI values of strain Pla85_3_4^T^ yielded maximal similarities ranging from 48.1 to 53.8% (Fig. 2), which also fall below the cut-off range of 60–80% for defining genera (Luo et al. 2014). The maximal similarities of a 1200 base pair region of the *rpoB* gene between strain Pla85_3_4^T^ were found to be in a range of 66.9–72.3% (Fig. 2), which again fall below the cut-off range of 75.5–78% for delineating genera (Bondoso et al. 2013; Kallscheuer et al. 2019d). Taken together, all four parameters support the conclusion that strain Pla85_3_4^T^ belongs to a novel genus. A most closely related genus could not be clearly identified, although this strain formed a branch with *Pirellula staleyi* in the MLSA-based tree.

### Morphological, physiological and biochemical analyses

**Fig. 3:**
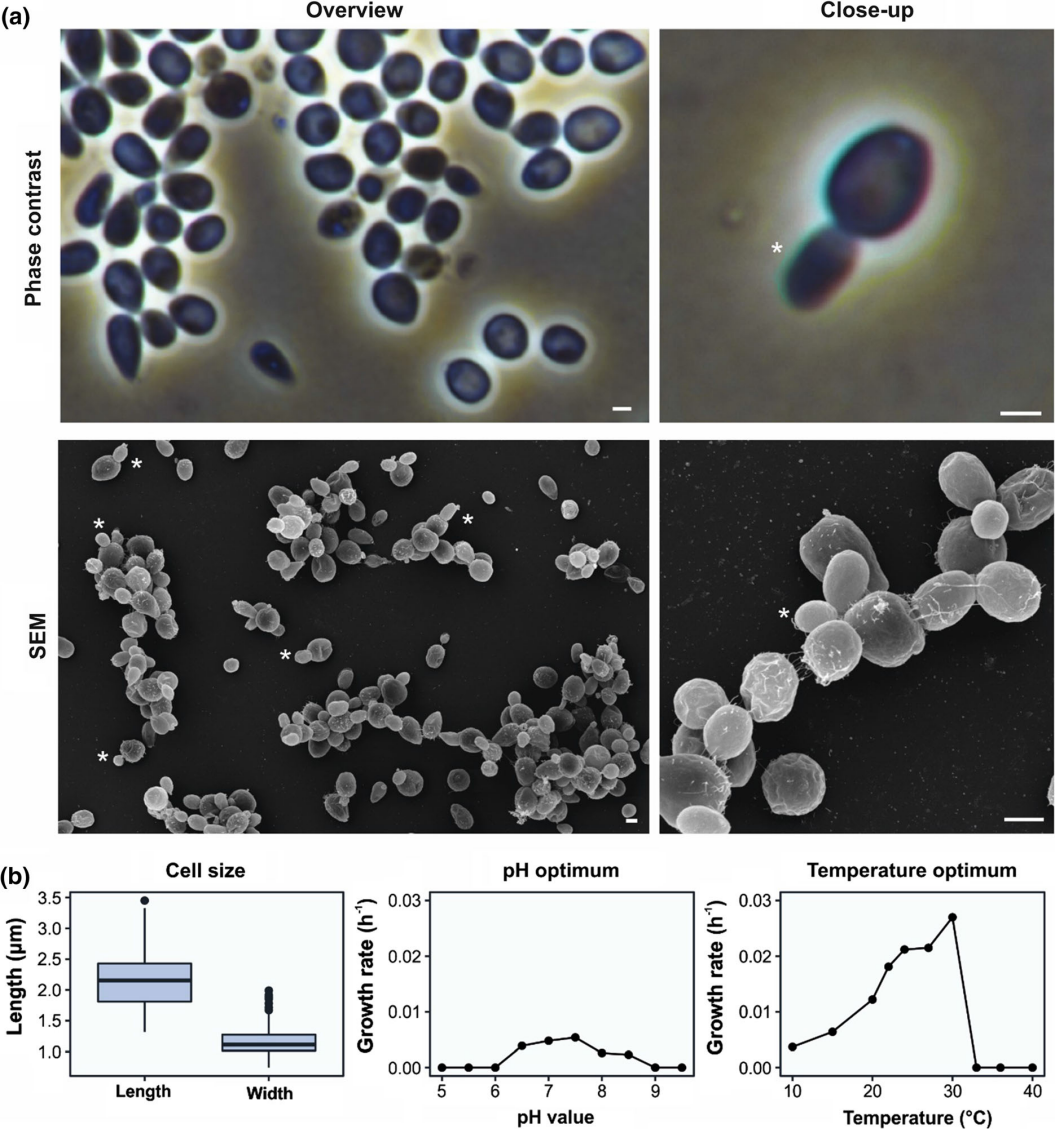
Phase contrast micrographs of strain Pla85_3_4^T^ (**a**) and cell size, pH optimum and temperature optimum (**b**). The strain grows in aggregates or rosettes and divides by polar budding, as can be observed in the overview and close up, respectively. Asterisks indicate budding cells. Scale bar is 1 µm

Morphology, physiology and life cycle of strain Pla85_3_4^T^ were found to be similar to those of many other Planctomycetes. Adult cells were attached by loose fimbriae, enabling the cells to grow in aggregates (Fig. 3a). In other Planctomycetes, these fimbriae originate from crateriform structures, but these structures were either absent or difficult to observe in this strain. When examined with phase contrast microscopy, the cells appeared round to pear-shaped and 2.2 ± 0.4 by 1.2 ± 0.3 µm in length and width, respectively (Table 1). As typical for members of the family *Pirellulaceae*, the cells divide by polar budding. Colonies of strain Pla85_3_4^T^ have a cream colour, indicating a lack of carotenoid production. The novel strain might be interesting for future studies on pigmentation of Planctomycetes since its phylogenetic position is between genera with mostly pigmented species (*Rhodopirellula*, *Rubripirellula*, *Novipirellula*, *Crateriforma*, *Roseimaritima*) and unpigmented species of the genera *Pirellula*, *Blastopirellula* and *Bremerella* (Fig. 1).

**Table 1:** Phenotypic and genotypic information of strain Pla85_3_4^T^ compared to members of the family *Pirellulaceae*

Characteristics	Pla85_3_4	*Bremerella cremea* LHWP2*	*Mariniblastus fucicola* FC18**	*Crateriforma conspicua* Pan14r***	*Pirellula staleyi* DSM 6068****	*Rubripirellula tenax* Poly51*****
*Phenotypic characteristics*						
Shape	Pear-shaped to round	Ovoid	Round	Pear-shaped	Teardrop- to pear-shaped	Round grain rice-shaped
Aggregates	Yes	Yes	Yes	Rosettes	Yes	Yes
Division	Budding	Budding	Budding	Budding	Budding	Budding
Flagella	n.o.	Yes	n.o.	n.o.	Monotrichous polar	n.o.
Crateriform structures	n.o	Yes	Yes	At fiber pole	At reproductive pole	Polar
Fimbriae	Polar matrix or fibre	n.o.	n.o.	Polar matrix or fibre	Yes	Matrix or fibre
Capsule	n.o.	n.o.	n.o.	n.o.	n.o.	Yes
Bud shape	Like mother cell	Like mother cell	Like mother cell	Like mother cell	Like mother cell	Like mother cell
Budding pole	Polar	n.d.	Any	Polar	Polar	Polar
Stalk	n.o.	n.o.	n.o.	n.o.	n.o.	n.o.
Holdfast structure	n.o.	n.o.	n.o.	n.o.	Yes	n.o.
Size (µm)	2.2 × 1.2	0.6–1.5 × 0.6–1.4	1.6–2.0	1.8 × 0.9	0.5–1.0 × 0.5–1.0	1.4 × 0.9
Colony colour	White/cream	White/cream	Light pink	Pink	White	Pink
*Genomic characteristics*						
Transposable elements	27	12	5	2	0	6
Transposable elements/Mb	2.82	1.91	0.76	0.28	0	0.75
Total genes	7178	5222	5200	5490	4767	6404
Genes/Mb	750	830	791	769	769	802
Giant genes	1	0	7	7	1	8
All proteins	7010	5145	5123	5400	4705	6274
Proteins/Mb	733	818	780	757	759	785
Hypothetical proteins	3033	3342	2087	2080	2601	2796
tRNAs	144	71	65	82	49	120
tRNAs/Mb	15.05	11.29	9.89	11.49	7.91	15.02
16S rRNA genes	1	1	1	1	1	1
Completeness (%)	98.28	98.28	98.28	98.28	98.28	98.28
Contamination (%)	5.17	1.72	1.72	0	0	1.72
Genome size (bp)	9,565,229	6,287,921	6,570,840	7,137,949	6,196,199	7,988,747
G + C content (%)	61.4	54.0 ± 2.6	53.4	57.8 ± 2.5	57.5	56.2 ± 2.1
Coding density (%)	85.3	86.8	88.8	88	86.2	88.8

*n.o.* not observed

*n.d.* not determined

*Lee et al. (2013), **Lage et al. (2017), ***Peeters et al. (2019), ****Schlesner and Hirsch (1984), *****Kallscheuer et al. (2019b)

Strain Pla85_3_4^T^ is able to grow in medium containing artificial seawater, consistent with the observation that the section of the river from which this strain was isolated is quite brackish due to the influx of water from the Baltic Sea. Strain Pla85_3_4^T^ grows chemoorganotrophically, aerobically and at temperatures ranging from 10 to 30 °C, with the optimum at 26 °C (Fig. 3b). The strain grows in the neutral to alkaline pH range from 6.5 to 8.5, with the optimum at 7.5. The growth rate of this strain was calculated to be 0.027 h^−1^, which corresponds to a doubling time of 26 h.

### Genomic characteristics

The genome of strain Pla85_3_4^T^ has a 61.4% G + C content and is 9,565,229 bp in length (Table 1). This distinguishes the strain from other members of the family *Pirellulaceae* as these species have smaller genomes of between 6.1 and 8.0 Mb, and G + C contents between 53.4 and 57.8%. The strain has a larger genome and a larger number of genes, while the coding density and number of proteins per Mb is lower than in its relatives from the family *Pirellulaceae* (Table 1). The genome of strain Pla85_3_4^T^ contains both a large number of transposable elements (27) and a large number of tRNAs (144) in comparison to those of other members of the family *Pirellulaceae* (Table 1). The genome of strain Pla85_3_4^T^ contains a singular 16S rRNA gene.

### Genome-based analysis of metabolic features

Based on the genome of strain Pla85_3_4^T^ and of species of closely related genera, we analysed the numbers of putative carbohydrate-active enzymes and of gene clusters putatively involved in the synthesis of secondary metabolites (Table 2). These numbers can give a first impression on the metabolic capabilities of the strain, e.g. in competitive environments, in which complex polysaccharides (e.g. derived from macroscopic phototrophs) function as a major source of carbon and energy. The observed number of 124 putative carbohydrate-active enzymes of strain Pla85_3_4^T^ is in the lower to middle range compared to its relatives, which harbour between 87 and more than 200 of such enzymes. Higher numbers of putative carbohydrate-active enzymes are not reflected by larger genomes. Strain Pla85_3_4^T^ has the largest genome of the compared strains, but is the strain with the second-lowest number of carbohydrate-active enzymes. Although the difference in the genome size of *Crateriforma conspicua* and *P. staleyi* is only around 1 Mb, the number of carbohydrate-active enzymes is nearly 2.5-fold different in a direct comparison of these two species. The numbers of proteins belonging to such classes are more likely a reflection of the complexity of the natural environment and probably do not depend on the genome size.

**Table 2:** Numbers of carbohydrate-active enzymes and putative gene clusters involved in the production of secondary metabolites in strain Pla85_3_4^T^ and close relatives

Metabolic feature	Pla85_3_4^T^	*Roseimaritima ulvae* UC8^T^	*Mariniblastus fucicola* FC18^T^	*Crateriforma conspicua* Mal65^T^	*Pirellula staleyi* DSM 6068^T^	*Rhodopirellula baltica* SH1^T^
Genome size (Mb)	9.57	8.21	6.57	7.18	6.20	7.15
*Carbohydrate-active enzymes*						
Glycoside hydrolase family	48	45	44	121	19	51
Glycosyltransferase family	46	76	57	65	45	64
Polysaccharide lyase family	6	3	5	7	1	6
Carbohydrate esterase family	9	7	5	9	8	12
Carbohydrate-binding module Family	15	21	15	15	14	10
Total	124	152	126	217	87	143
*Secondary metabolite clusters*						
Terpenoid	3	2	2	2	3	2
Type I PKS	1	3	0	2	0	2
Type II PKS	0	0	0	0	0	0
Type III PKS	1	1	0	0	0	1
Type I PKS-NRPS	1	1	2	2	0	1
NRPS	0	0	0	1	0	0
Bacteriocin	3	0	0	0	1	0
Ectoine	1	0	0	0	0	0
Resorcinol	1	0	0	0	0	0
Total	11	7	4	7	4	6

In contrast, numbers of gene clusters putatively involved in the production of secondary metabolites clearly correlated with the genome size. Strain Pla85_3_4^T^ has both the largest genome and the highest number of predicted clusters (Table 2). The lowest numbers were found in *Mariniblastus fucicola* and *P. staleyi*, both with genomes below 7 Mb. In strain Pla85_3_4^T^, three clusters putatively involved in the biosynthesis of bacteriocin, one cluster for ectoine production and one cluster related to resorcinol production were identified. These appear to be restricted to strain Pla85_3_4^T^ (of the other strains examined only *P. staleyi* harbours one putative cluster related to bacteriocin production). Three terpenoid biosynthetic gene clusters are present in Pla85_3_4^T^, however, since the strain lacks pigmentation, they are likely not exclusively involved in carotenoid biosynthesis, but relevant for synthesis of other terpenoids. Putative polyketide synthases of the types I and III are present in all strains, except for the two above-mentioned species with genome sizes smaller than 7 Mb. Type II polyketide synthases appear to be absent from all compared strains.

## Conclusion

Taken together, based on the phylogenetic inference and supported by differences in morphology as well as genomic characteristics, we conclude that strain Pla85_3_4^T^ represents a novel species of a new genus, for which we propose the name *Lignipirellula cremea* gen. nov., sp. nov.

### *Lignipirellula* gen. nov.

*Lignipirellula* (Lig.ni.pi.rel’lu.la. L. neut. n. lignum wood; N.L. fem. n. *Pirellula* name of a bacterial genus; N.L. fem. n. Lignipirellula a *Pirellula* isolated from wood).

Members of the genus have a Gram-negative cell envelope architecture, are aerobic, mesophilic, neutrophilic and heterotrophic. Cells are round to pear-shaped and divide by polar budding. The genus is part of the family *Pirellulaceae*, order *Pirellulales*, class *Planctomycetia*, phylum *Planctomycetes*.

### *Lignipirellula cremea* sp. nov.

*Lignipirellula cremea* (cre’me.a. N.L. fem. adj. *cremea* of creme; corresponding to the creamy colour of the cells).

In addition to the characteristics described for the genus, cells have a length of 2.2 ± 0.4 µm and width of 1.2 ± 0.3 µm, form aggregates and lack pigmentation. Crateriform structures are not observed. Daughter cells have the same shape as the mother cell. Grows aerobically at 10–30 °C (optimum 26 °C) and at pH 6.5–8.5 (optimum 7.5), and has a doubling time of about 26 h. The 9.6 Mb genomic DNA of the type strain has a G + C content of 61%.

The type strain is Pla85_3_4^T^ (DSM 103796^T^ = LMG 29741^T^), which was isolated from the surface of wood incubated close to the discharge of a wastewater treatment plant in the Unterwarnow river in Rostock, Germany in September 2014. The genome (accession no. CP036433) and 16S rRNA sequence (GenBank accession no. MK559988) of strain Pla85_3_4^T^ are available from GenBank.
